# 2483: Evaluating a community research consult service: Getting to outcomes and impacts

**DOI:** 10.1017/cts.2017.283

**Published:** 2018-05-10

**Authors:** Clara Pelfrey, Katrice Cain, Mary Ellen Lawless, Earl Pike, Ashwini Sehgal

**Affiliations:** Case Western Reserve University, Cleveland, OH, USA

## Abstract

OBJECTIVES/SPECIFIC AIMS: This study describes the design, operation, and evaluation of a community-based research (CBR) consult service within the setting of a Clinical and Translational Science Award (CTSA) institution. To our knowledge, there are no published evaluations of a CBR consult service at a CTSA hub. METHODS/STUDY POPULATION: A CBR consult service was created to support faculty, healthcare providers/research coordinators, trainees, community-based organizations, and community members. A framework was developed to assess the stages of client engagement and to foster clear articulation of client needs and challenges. A developmental evaluation system was integrated with the framework to track progress, store documents, continuously improve the consult service, and assess research outcomes. RESULTS/ANTICIPATED RESULTS: This framework provides information on client numbers, types, services used, and successful outreach methods. Tracking progress reveals reasons that prevent clients from completing projects and facilitates learning outcomes relevant to clients and funding agencies. Clients benefit from the expert knowledge, community connections, and project guidance provided by the consult service team, increasing the likelihood of study completion and achieving research outcomes. DISCUSSION/SIGNIFICANCE OF IMPACT: Our evaluation suggests that clients benefit by (1) gaining the collective knowledge of the experts comprising the team, (2) learning the process of doing CBR, including the required steps to reach completion, and (3) gaining a project management mentality promoting translational research outcomes. This study offers a framework by which CTSA institutions can expand their capacity to conduct and evaluate CBR while addressing challenges that inhibit community engagement.

